# Clinical and immunological characteristics of rheumatic diseases in children

**DOI:** 10.1186/1546-0096-12-S1-P211

**Published:** 2014-09-17

**Authors:** Iryna Chyzheuskaya, Ludmila Belyaeva, Helena Hrustaleva, Helena Kolupaeva, Rostislav Filonovich, Larisa Zaitseva, Tamara Yuraga

**Affiliations:** 1 Belarusian medical academy of postgraduate education, Minsk, Belarus; 2 4 City Children's Clinical Hospital , Minsk, Belarus

## Introduction

The problem of rheumatic diseases (RD) in childhood is associated with increasing prevalence, severity and frequency of adverse events and complications. RD group of children are more common juvenile idiopathic arthritis (JIA), systemic lupus erythematosus (SLE) and juvenile scleroderma (JS).

## Objectives

To investigate the clinical and immunological response and the role of infectious factors in the development of RD in children.

## Methods

60 patients with RD were examined in the 4th City Children's Hospital in Minsk (31 children with JIA, 16 with JS, 13 with SLE). To identify organ pathology used general clinical research methods, including a complex of functional, instrumental and laboratory diagnostic tests, including the determination of levels of TNF-α and IFN-γ.

## Results

20 patients with JIA had an articular form, 11 children had an systemic form. 2 patients had diffuse form of systemic sclerosis (SS). 6 patients had a limited form of SS. 8 children had scleroderma. 8 children with JS, 13 children with JIA and all children with SLE had fever, weakness, weight loss at the onset of the disease. 3 children with JS, 12 children with JIA and 5 with SLE had lymphadenopathy. The most of children had in the blood serum IgG antibodies to herpes simplex virus (12 with JIA, 10 with JS, 6 with SLE), Epstein-Barr virus (8 with JIA, 3 with JS, 2 with SLE) and cytomegalovirus (6 with JIA, 3 with JS), IgG to Borrelia burgdorferii (17 with JIA, 9 with JS), to Chlamidia psittacii (10 with JIA). It can be assumed that these organisms can act as triggers for the development of this pathology. The same changes, characterized by a primary decrease CD8+-cells against a background of normal or elevated content of CD4+-cells been established in children with RD. Individual values of TNF-α in serum were elevated in 8 (88.9%) patients with JS, in 5 (62.5%) children with SLE and in 11 (84.6%) children with JIA. The positive relationship of TNF-α content with the content of CRP (r=0.64; P<0.01). The content of TNF-α was significantly higher in patients with high values of rheumatoid factor (RF) than in children with normal levels of RF (P <0.05). According to the results of the research content of IFN-γ in the serum of all patients with RD level of IFN-γ was significantly reduced compared with healthy children (P <0.05).

## Conclusion

Revealed changes in the immune system of children with RD characterized by an imbalance of immunoregulatory subpopulations of T-lymphocytes, cytokine imbalance with pro-inflammatory and anti-inflammatory functions in conjunction with persistent viral and bacterial infection, are the basis for immunotherapy in treatment of children with RD.

## Disclosure of interest

None declared.

